# News Items

**Published:** 1886-02

**Authors:** J. A. Wessinger

**Affiliations:** Secretary, Howell, Michigan


### News Items.

A Sanitary Convention will be held at Howell, Michigan, under the auspices of the State Board of Health, on Wednesday and Thursday, March 3 and 4, 1886.

There will be sessions the first day at 2:00 P. M. and 7:30 P. M.; on the second day at 9:30 A. M., 2 P. M. and 7:30 P. M., standard time.

At each session of the Convention there will be addresses or papers on subjects of general interest pertaining to public health, each paper to be followed by a discussion of the subject treated.

The President of the Convention will be the Rev. M. H. Pettit.

Vice Presidents: Hon. William McPherson, Jr., Howell; L. S. Montague, Esq., Howell; R. H. Person, Esq., Howell; Hon. Jay Corson, Howell; Hon. Neil O'Hern, Charles G. Jewett, Judge Arthur E. Cole, Dr. W. L. Wells, Dr. Robert B. Bell, Hon. E. B. Winans, Hon. William Ball, Hamburg; E. J. Hardy, Esq., Oceola; Fred. H. Warren, Esq., Fowlerville; Hon. George Coleman, Marion; Dr. C. W. Haze, Pinckney; Dr. J. J. Boyd, Hartland; Dr. W. H. Erwin, Oak Grove; Dr. W. J. McHench, Brighton.—Secretary, Dr. J. A. Wessinger.

Invitation is especially extended to health-officers to be present and take part in the discussions.

The objects of the Convention are the presentation of facts, the comparison of views, and the discussion of methods relating to the prevention of sickness and death, and the improvement of the conditions of living.

ADDRESSES AND SUBJECTS TO BE PRESENTED AND DISCUSSED.

Welcoming address by Hon. Jay Corson, Mayor of Howell.

An address by the President of the Convention, Rev. M. H. Pettit.

Among the subjects which it is expected will be presented and discussed are the following:—

1. The sanitary condition and needs of school buildings and grounds, and of other public buildings and grounds in Howell.

2. The general subject of water-supply, and the present condition of the water-supply in Howell, the depth of wells and the details of their surroundings.

3. The disposal of slops, garbage, refuse, etc.

4. The prevention of communicable diseases.

5. Ventilation.

6. The drainage and sewerage of Howell.

The papers are expected to be original contributions, which when read are to be considered the property of the Convention, and to be left with the Secretary. Programmes will be issued before the Convention.

The Committee from the State Board of Health consists of J. H. Kellogg, M. D., Battle Creek, and Henry B. Baker, M. D., Lansing.

DR. J. A. WESSINGER, *Secretary*,
Howell, Michigan.

